# HIV/AIDS in Pakistan: the battle begins

**DOI:** 10.1186/1742-4690-4-22

**Published:** 2007-03-21

**Authors:** Mohammad A Rai, Haider J Warraich, Syed H Ali, Vivek R Nerurkar

**Affiliations:** 1 Biological and Biomedical Sciences, Aga Khan University, Karachi, Pakistan; 2 Retrovirology Research Laboratory, Department of Tropical Medicine, Medical Microbiology and Pharmacology, Asia-Pacific Institute of Tropical Medicine and Infectious Diseases, John A. Burns School of Medicine, University of Hawaii, Honolulu, Hawaii, USA

## Abstract

Pakistan, the second most populous Muslim nation in the world, has started to finally experience and confront the HIV/AIDS epidemic. The country had been relatively safe from any indigenous HIV cases for around two decades, with most of the infections being attributable to deported HIV positive migrants from the Gulf States. However, the virus finally seems to have found a home-base, as evidenced by the recent HIV outbreaks among the injection drug user community. Extremely high-risk behavior has also been documented among Hijras (sex workers) and long-distance truck drivers. The weak government response coupled with the extremely distressing social demographics of this South-Asian republic also helps to compound the problem. The time is ripe now to prepare in advance, to take the appropriate measures to curtail further spread of the disease. If this opportunity is not utilized right now, little if at all could be done later.

## Introduction

Pakistan, the world's second most populous Muslim nation, has started to finally experience and confront the HIV/AIDS epidemic. Largely portrayed as having free of this menace till now, this South-Asian republic seems to be following in suit with its HIV-havocked neighbor, India. With isolated outbreaks being reported all over the country, time already seems to be running out for the sixth most populous country in the world.

## Evolution

The first reports of HIV in Pakistan in 1987 implicate contaminated blood transfusions [1] as one of the culprits. The other route alludes to expatriates or Pakistanis settled abroad. These seem to be the more important risk factor for acquisition of HIV, as demonstrated amply by the fact that around 70% of the total positive HIV cases from a sample of over 15,000 individuals over a period of six years (1986–1992) fell into this category [2]. The bulk of the infected were deported workers from the Gulf States [3]. Pakistan, as compared to its neighbors, has remained relatively safe from any indigenously acquired cases of HIV for about two decades. The situation however changed in 2004 when Pakistan experienced its first full-fledged HIV outbreak [4]. In the remote desert town of Larkana, the HIV bubble-burst took place amongst the injection drug user (IDU) community. What this basically meant was that the virus had finally found a home-base, as evidenced later by outbreaks all over the nation [5].

## High-risk Populations

The HIV/AIDS epidemic in Pakistan is following along the same atypical lines as it has done so far in the rest of Asia. Starting from isolated high-risk population subgroups, the virus jumps the barrier to cross into the mainstream general populace. Once this barrier is crossed, little if at all anything can be done to prevent a complete HIV onslaught.

Similar to its south-east Asian neighbors, the greatest risk for the spread of HIV in Pakistan stems from IDU. Currently estimated at over 180,000 in number [6], the ongoing strife in Afghanistan, the worlds largest poppy producing country, seems only to swell up this number even more in the future [7]. IDU all over the country have started recording alarmingly high rates of HIV. According to the latest figures released by the National AIDS Control Program of Pakistan, HIV/AIDS prevalence among IDUs has jumped from 0.4% in December 2003 to 7.6% in 2004. However, in Larkana, where Pakistan's first HIV outbreak among IDU was reported, the number approached an astounding, twenty-seven percent [4]. After the Larkana episode, HIV has been documented among IDU all over Pakistan. Currently, IDU do not comprise the bulk of drug users in Pakistan [8]. The number of IDU is bound to increase in the near future, and as this happens, the relative cases of HIV/AIDS will also rise. The first hurdle in the spread of HIV seems to be already traversed.

Sex workers in Pakistan represent the second most serious threat for HIV transmission. The government refuses to accept illicit sex underway in the country, although there are established prostitution centers in all the major cities of Pakistan. The so-called 'red-light' areas, in addition to female prostitutes, also house Hijras – male transvestites. These Hijras provide valuable insight into HIV demographics, as data pertaining to female commercial sex workers is very limited. Reports [5] suggest that the HIV prevalence among Hijras in Karachi, a city of 13 million people in southern Pakistan, approximates around 4%. The situation is bound to be even worse in the rural parts, particularly in the Pathan-dominated northern Pakistan, where homosexuality is socially tolerated [9]. The majority of men having sex with men in Pakistan are married [10], which brings into light their possible potential as acting as a bridge to the general population.

Truck drivers are also a very important subgroup, primarily because of their role in fuelling the HIV epidemic in neighboring Madras, India [11]. In a survey done in Lahore, Pakistan's central Hub for long-distance truckers, over 49% of them reported having sex with another man [12]. The possibility of horizontal ellipsis across the border from India has also been raised [13].

Once the high-risk populations have acquired the virus, it is only a matter of time before the general populace falls prey to it. IDU, commercial sex workers, truck drivers, etc., facilitate in bridging this gap. What is alarming is the fact that once the virus moves from the urban population to the rural population, the effect will be much more catastrophic, not only because the bulk of the Pakistani population resides here (only 34% lives in urban areas) [17] but also due to almost non-existent healthcare-facilities.

## Steps Underway

Decades of corruption and poor planning of resources have translated into a fight for Pakistan's very own continued existence. Keeping this in mind and the horde of other problems currently encountering Pakistan, any efforts directed towards prevention and control of HIV/AIDS are quite laudable.

The bulk of the credit in this regard goes to the private sector. Over 50 non-governmental organizations (NGO) are working to improve the HIV/AIDS status quo in Pakistan [5]. Their work ranges from providing needle-exchange programs for IDU to spreading awareness about HIV/AIDS to the masses. Worth mentioning is the organization, 'AMAL,' which means 'action' in Pakistan's national language, Urdu. It has outreach HIV training programs focusing not only on IDU but also for the out-of-the-limelight population, female sex workers.

On the other side, the current government policy falls under the auspices of the National HIV/AIDS Strategic Framework. The program has four foci: improved HIV prevention, expanding interventions among vulnerable groups, preventing transfusion related infections and improving infrastructure [15]. With over Rs. 2.9 billion (US $48 million) at its disposal, the program hopefully would chalk out a practical, concrete plan and then initiate work to implement it.

## The Social Demographics

It may sound ludicrous but the fact remains that to properly combat any problem, the affected have to first accept it and then conquer over it. The society in Pakistan has as yet not accepted HIV/AIDS as having anything to do with them. Trends may be changing, but the age-old stigmas and taboos related to HIV still persist. HIV is considered extremely shameful, particularly in the rural setting. Even discussions on this topic are frowned upon. Awareness about HIV/AIDS in general is extremely limited. The severity of the situation could be deduced from a survey conducted among school teachers in the capital city, Islamabad. An outstanding sixty percent of the teachers responded by saying that 'they thought HIV was irrelevant in our cultural setting.' [16] This awareness and acceptance issue would indeed be a big challenge, because 'teachers' as well as 'children' will need to be taught.

UNAIDS latest figures estimate the number of cases in Pakistan bordering 85,000 [14]. Underreporting and limited surveillance means that the actual number of infected is much higher. Keeping in mind the poor healthcare facilities, the appallingly low literacy rate (in 2001, the illiteracy rate for Pakistani women over 15-year old was 72%) [17], and a mushrooming population (growth rate of Pakistan lies at 2.5%) [17], the stakes for a battle against HIV are indeed very low.

## Conclusion

The situation concerning Pakistan and HIV is indeed very precarious. The country lies at a very crucial junction. HIV has as yet not exploded. Most of the populace remains safe, as for now. However, concentrated epidemics have emerged, which means that very little time is left before a steep rise in infections occurs. The battle against HIV/AIDS in Pakistan has to be fought on a number of fronts: not just the afflicted population, but also on changing peoples' perspectives and ushering in the proper government policies and response measures. Neighboring China serves as a good example to follow as regards formulation of a national policy about HIV/AIDS [18]. The Government has to come forward and face the truth about HIV in Pakistan. Embarking not only upon national-level mass awareness programs, practical steps including wide-spread screening for the high-risk populations has also to be instituted. Stigma and discrimination about HIV/AIDS in society could only be removed when prominent figures including politicians and sport stars start discussing about HIV/AIDS in public. As soon as this stigmatization barrier is overcome, a major chunk of the battle against HIV in Pakistan would be conquered. What has to be reiterated again is that the time to act is now. Timely steps taken at the present can go a long way in preventing a wide-spread HIV epidemic in Pakistan.
